# Short-term glutamine supplementation decreases lung inflammation and the receptor for advanced glycation end-products expression in direct acute lung injury in mice

**DOI:** 10.1186/1471-2466-14-115

**Published:** 2014-07-15

**Authors:** Yin-Ching Chuang, Huey-Mei Shaw, Chi-Chung Chen, He-Jia Pan, Wei-Chih Lai, Hui-Ling Huang

**Affiliations:** 1 Department of Medicine, Chi Mei Medical Center-Liou Ying, Tainan, Taiwan; 2 Department of Health and Nutrition, Chia-Nan University of Pharmacy and Science, Tainan, Taiwan; 3 Department of Medicine, Medical Research, Chi Mei Medical Center, Tainan, Taiwan

**Keywords:** Glutamine (GLN), Acute lung injury (ALI), Receptor for advanced glycation end-products (RAGE), Inflammation

## Abstract

**Background:**

Glutamine (GLN) has been reported to improve clinical and experimental sepsis outcomes. However, the mechanisms underlying the actions of GLN remain unclear, and may depend upon the route of GLN administration and the model of acute lung injury (ALI) used. The aim of this study was to investigate whether short-term GLN supplementation had an ameliorative effect on the inflammation induced by direct acid and lipopolysaccharide (LPS) challenge in mice.

**Methods:**

Female BALB/c mice were divided into two groups, a control group and a GLN group (4.17% GLN supplementation). After a 10-day feeding period, ALI was induced by intratracheal administration of hydrochloric acid (pH 1.0; 2 mL/kg of body weight [BW]) and LPS (5 mg/kg BW). Mice were sacrificed 3 h after ALI challenge. In this early phase of ALI, serum, lungs, and bronchoalveolar lavage fluid (BALF) from the mice were collected for further analysis.

**Results:**

The results of this study showed that ALI-challenged mice had a significant increase in myeloperoxidase activity and expression of interleukin (IL)-1β, IL-6, and tumor necrosis factor-α in the lung compared with unchallenged mice. Compared with the control group, GLN pretreatment in ALI-challenged mice reduced the levels of receptor for advanced glycation end-products (RAGE) and IL-1β production in BALF, with a corresponding decrease in their mRNA expression. The GLN group also had markedly lower in mRNA expression of cyclooxygenase-2 and NADPH oxidase-1.

**Conclusions:**

These results suggest that the benefit of dietary GLN may be partly contributed to an inhibitory effect on RAGE expression and pro-inflammatory cytokines production at an early stage in direct acid and LPS-induced ALI in mice.

## Background

Acute respiratory distress syndrome (ARDS) is often seen in critically ill patients, and increases their risk of secondary bacterial pneumonia due to direct gastric fluid reflux, bacterial pneumonia, and systemic sepsis [1]. Inflammatory cytokines can recruit inflammatory cells from the blood and increase the permeability of the alveolar–capillary membrane after an acute lung injury (ALI) challenge [2]. ALI is characterized by significant oxidative stress and inflammation in the lung, which can contribute to cellular injury [3,4].

The receptor for advanced glycation end-products (RAGE) plays a critical role in the pathogenesis of direct acid or lipopolysaccharide (LPS)-induced ALI, and obstruction of RAGE signaling has a protective effect against ALI [5]. RAGE was markedly increased in bronchoalveolar lavage fluid (BALF) in a dose-dependent manner in direct acid (pH 1.0)-induced ALI [6]. RAGE has been implicated as an early mediator of ALI, similar to other cytokines such as tumor necrosis factor (TNF)-α or interleukin (IL)-1β [6,7]. RAGE is considered a useful indicator of early stage ALI in alveolar epithelial type I cell injury, which correlated with the severity of lung damage [8]. RAGE–ligand interactions could result in the activation of nuclear factor (NF)-κB and caused subsequent serial immune–inflammatory processes [9].

Additionally, a previous report indicated that enhanced oxidative stress was often observed in endotoxin-induced ALI [3]. Selective inducible nitric oxide synthase (iNOS) inhibition by a NOS inhibitor was effective in reducing TNF-α, IL-6, and nitrite/nitrate levels in BALF by acid aspiration in rats [10]*.* Antioxidant N-acetylcysteine (NAC) treatment protected the lungs from stress in the first 12 h after lipopolysaccharide (LPS) administered intratracheally (IT) and also decreased the mRNA expression of NF-κB, IL-6, TNF-α, and cyclooxygenase (COX)-2 inhibitors [11]. Thus, inhibition of oxidative stress is considered a potential strategy for ameliorating direct lung injury by LPS or acid challenge.

Glutamine (GLN) is a conditionally essential amino acid during critical illness and injury. GLN has been shown to improve clinical and experimental sepsis outcomes, owing to its anti-inflammatory and antioxidative properties [12,13]. Results of recent studies indicate that a single intravenous dose of GLN supplementation ameliorated lung and kidney injury and increased survival in a cecal ligation and puncture (CLP)-induced sepsis model by inhibition of the high-mobility group box protein-1 (HMGB-1)-mediated pathway, which was thought to be a late-stage mediator of systemic damage [14,15]. However, the mechanisms underlying the actions of GLN remain undefined, and may depend on the route of administration and the model of ALI used.

To date, no study had definitively described the effects of dietary GLN on IT administration of acid and LPS induced-ALI in mice. We hypothesized that GLN may have anti-inflammatory effects on a mouse model of direct acid and LPS-induced ALI. To test this idea, the mRNA and protein expression of proinflammatory cytokines and RAGE were measured. We aimed to determine whether short-term GLN supplementation has a protective effect in the early stages of injury.

## Methods

### Animals and diets

Six-week-old female BALB/c mice (body weight [BW], 17–20 g), purchased from the Laboratory Animal Center of BioLASCO (Taipei, Taiwan), were housed individually in stainless steel wire cages in a temperature- and humidity-controlled room supplied with a regular 12-h light/dark cycle. All mice were allowed free access to a chow diet and tap water for 7 days before the study. The protocols for animal care and handling were approved by the Institutional Animal Care and Use Committee of the Chia-Nan University of Pharmacy and Science (CN-IACUC-101028).

Mice were randomly assigned to a control group (n = 31) and a GLN group (n = 31). Table 1 shows the composition of the two test diets. Both diets were designed according to the AIN-93G formula, which was modified to contain 15% soybean oil [16]. For the GLN diet, 4.17% GLN was added to the formula, replacing casein and providing 25% of total amino acid nitrogen. The dose of GLN was based on previous studies indicating that this dosage of GLN had beneficial immunomodulatory effects [17,18]. Both diets were isonitrogenous and isocaloric. All mice were fed the test diets for 10 days. Finally, 10 mice from the control and GLN groups were sacrificed as unchallenged controls, and then the remainder was given the ALI challenge. After ALI challenge, nine mice in both groups were used for survival rate assessment. Twelve mice in both groups were killed with intraperitoneal injections of sodium pentobarbital (50 mg/kg) after 3 h of ALI challenge. For survival studies, an analgesic was not provided after ALI challenge. All of the mice were housed individually in a quiet, comfortable environment after ALI challenge. Then, the mice were observed once every h for occurrence of mortality.

**Table 1:** **Composition of test diets**^**a**^

	**Control**	**GLN**
	**(g/kg diet)**	
Corn starch	449.5	457.8
Casein	200	150
L-glutamine (GLN)^b^	-	41.7
Sucrose	100	100
Soybean oil^a^	150	150
Alphacel non-nutritive bulk (fiber)	50	50
AIN-93G mineral mixture	35	35
AIN-93G vitamin mixture	10	10
L-Cystine	3	3
Choline bitartrate	2.5	2.5
2,5-di-tert-Butylhydroquinone	0.014	0.014
% Energy^c^		
Carbohydrate	50.6	50.6
Protein	18.4	18.4
Fat	31	31

^a^Our test diets were 15% high fat diets that modified from the AIN-93G standard formula.

^b^The dose of GLN supplement was provided 25% of total amino acid nitrogen. Both of diets were isonitrogenous.

^c^The two diets were isocaloric.

### Acid and LPS-induced ALI model

After 10 days of feeding, 21 mice in both groups were weighed and anesthetized with sodium pentobarbital (intraperitoneal [IP] injection) before inducing ALI by acid and LPS aspiration. The procedure for IT instillation was described in a previous report [19]. HCl (pH 1.0, in sterile distilled water; 2 mL/kg BW) was administered IT to each mouse. After 5 min, LPS from *Escherichia. coli* (serotype 055:B5, Sigma Aldrich Co, LLC, St. Louis, MO, USA) was instilled IT (10 μg in 50 μL sterile saline; 5 mg/kg BW) [6,10]. Twelve mice in both groups were sacrificed with IP injections of sodium pentobarbital (50 mg/kg) 3 h after ALI challenge.

### Tissue sampling and preparation

Blood was collected via the abdominal vena cava, and the lung tissue was excised and weighed. The blood was centrifuged at 3500 × g at 4°C for 10 min and the serum was stored at -80°C for future total antioxidant system (TAS), TNF-α, and IL-6 analysis. A small piece of lung tissue (approximately 30 mg) was excised, placed in storage reagent (RNAfter, GeneMark Ltd, Tainan, Taiwan), and stored at -80°C for RNA extraction and subsequent real-time polymerase chain reaction (PCR) analysis. The remaining sample was homogenized in an ice-cold potassium phosphate (KP) buffer (0.01 M; pH 7.4) containing 1.15% KCl, using a Potter–Elvehjem-type homogenizer with a Teflon pestle. A portion of the lung homogenate was centrifuged at 15,000 × g at 4°C for 30 min, and the supernatant was taken as the post-mitochondrial fraction (PMS) for measuring the activity of antioxidative enzymes and for cytokine analysis. The pellets were resuspended in ice-cold KP buffer (50 mM phosphate buffer; pH 6.0) that contained 0.5% hexadecyltrimethylammonium bromide (HTAB), and were stored at -80°C for further myeloperoxidase (MPO) analysis.

### Determination of MPO activity

The method of MPO activity analysis was modified from that described in previous studies [20,21]. After snap freezing in liquid nitrogen and thawing for three cycles, samples were centrifuged at 14,000 × g at 4°C for 10 min. Aliquots of the supernatants were incubated in a 50 mM KP buffer containing 0.05% hydrogen peroxide and *o*-dianisidine dihydrochloride (0.167 mg/mL). MPO activity was measured by the change in absorbance at 460 nm over 3 min, using a spectrophotometric method. The protein content was determined using Lowry’s method [22].

### Serum TAS, lung glutathione, and antioxidative enzyme analysis

TAS was measured using commercially available enzyme-linked immunosorbent assay (ELISA) kits, in accordance with the manufacturer’s instructions (Cayman Chemical Co, Ann Arbor, MI, USA). Analysis was finally read at 450 nm in a spectrophotometer. Glutathione (GSH) content and glutathione peroxidase (GPx), glutathione reductase (GR), glutathione S-transferase (GST), superoxide dismutase (SOD), and catalase activity were measured as described elsewhere [23,24].

### BALF collection

Ten mice from both groups were used for BALF collection by bronchoalveolar lavage after ALI challenge. A 20-gauge catheter was inserted into each mouse’s trachea, and 1 mL sterile saline was flushed back and forth three times. The recovered lavage was then centrifuged at 15, 000 × g at 4°C for 10 min [19]. The supernatant aliquots were stored at -80°C for further analysis of cytokines and RAGE by ELISA.

### Quantification of cytokines and RAGE by ELISA

Levels of IL-1β, IL-6, TNF-α, and RAGE were measured using commercially available ELISA kits, in accordance with the manufacturer’s instructions (eBioscience, San Diego, CA USA and R&D Systems, Minneapolis, MN, USA). Each result was finally read at 450 nm in a spectrophotometer.

### RNA isolation and mRNA detection

Total RNA was extracted from the lung immersed in storage solution (RNAfter) using an illustra RNAspin Mini RNA Isolation Kit (GE Healthcare, Buckinghamshire, UK), according to the recommended protocol. The RNA quality was confirmed by a 28S ribosomal RNA:18S ribosomal RNA ratio of 2 after ethidium bromide staining. Levels of mRNA for COX-2, iNOS, IL-1β, IL-6, RAGE, and NADPH oxidase-1 (NOX-1), were determined by real-time PCR. Total RNA (1 μg) was reverse-transcribed into first-strand cDNA using 200 units of Moloney murine leukemia virus (MMLV-RT; Promega Corp, Madison, WI, USA). PCR was performed using 50 ng cDNA, 2 SYBR Green PCR Master Mix (Applied Biosystems, Foster City, CA, USA), and 200 nM of the primer pair. The sequences of the PCR primers used are shown in Table 2. Amplification using 40 cycles of two steps (95°C for 15 s and 60°C for 1 min) was performed on an ABI Prism 7300HT sequence detection system (Applied Biosystems, Foster City, CA, USA). To confirm the amplification of specific transcripts, melting curve profiles were produced at the end of each run. In this assay, glyceraldehyde-3-phosphate dehydrogenase (GAPDH) was used as an internal control. Each value was normalized to that of GAPDH, and then the relative mRNA abundance was calculated by taking the normalized value for the control group as 1.

**Table 2:** Sequences for real-time PCR primers and GenBank accession numbers

**Gene**	**GenBank accession**	**Amplicon size (bp)**	** Sequence**
**COX-2**	NM_011198	147	F: 5′-AAGCCGAGCACCTTTGGAG
			R: 5′-ATTGATGGTGGCTGTTTTGGTAG
**iNOS**	NM_010927	63	F: 5′-GGGCAGCCTGTGAGACCTT
			R: 5′-TGAAGCGTTTCGGGATCTG
**IL-1β**	NM_008361	99	F: 5′- GATCCCAAGCAATACCCAAA
			R: 5′- GGGGAACTCTGCAGACTCAA
**IL-6**	NM_031168	140	F: 5′- TCCAGTTGCCTTCTTGGGAC
			R: 5′- GTGTAATTAAGCCTCCGACTTG
**Nox-1**	NM_172203	215	F: 5′-CTGACAAGTACTATTACACGAGAG
			R: 5′- CATATATGCCACCAGCTTATGGAAG
**RAGE**	NM_007425	91	F: ACTACCGAGTCCGAGTCTACC
			R: CCCACCTTATTAGGGACACTGG
**GAPDH**	NM_008084	123	F: 5′-AGGTCGGTGTGAACGGATTTG
			R: 5′-TGTAGACCATGTAGTTGAGGTCA

F, Forward; R, Reverse; COX-2, cyclooxygenase-2; GAPDH, glyceraldehyde-3-phosphate dehydrogenase; iNOS, inducible nitric oxide synthase; IL-1β, interleukin 1β; IL-6, interleukin 6; Nox-1, NADPH oxidase; RAGE, advanced glycation end-products.

### Statistical analysis

The main effects of dietary GLN and ALI were evaluated by two-way analysis of variance (ANOVA). The differences among the four groups were analyzed by Duncan’s multiple range test. The significance of differences between two groups was analyzed by Student’s *t*-test. The statistical significance level was set at *p* < 0.05. All statistical analyses were performed using SAS software (SAS Institute Inc., Cary, NC, USA).

## Results

### Body weight gain, food intake, and lung weight

As shown in Table 3, ALI challenge reduced body weight gain and feeding efficiency. Food intake was not changed by GLN or ALI treatment. Lung weight and relative lung weight increased approximately 60% and 65% by ALI challenge, respectively. These results revealed that acid and LPS aspiration caused marked enlargement of the lung, but there was no effect of dietary GLN supplementation. GLN treatment had no adverse effect on growth in mice.

**Table 3:** **Body weight gain, food intake, feed efficiency, and lung weight of mice**^**a**^

	**Unchallenged**		**ALI**		***p *** **values from Two-way ANOVA**^**b**^		
	**Control**	**GLN**	**Control**	**GLN**	**ALI**	**GLN**	**ALI × GLN**
Initial body weight (g)	20.0 ± 0.9	20.0 ± 0.9	20.1 ± 0.8	20.1 ± 0.8	NS	NS.	NS.
Body weight gain (g/d)	0.25 ± 0.09	0.21 ± 0.17	0.19 ± 0.10	0.15 ± 0.11	< 0.05	NS.	NS.
Food intake (g/d)	4.24 ± 0.28	4.11 ± 0.30	4.50 ± 0.44	4.32 ± 0.84	NS.	NS.	NS.
Feed efficiency (%)^c^	5.89 ± 2.08	4.90 ± 4.36	4.06 ± 2.14	3.40 ± 2.49	< 0.05	NS.	NS.
Lung weight (g)	0.15 ± 0.03	0.14 ± 0.01	0.24 ± 0.06	0.23 ± 0.04	< 0.001	NS.	NS.
Relative lung weight (%)^d^	0.65 ± 0.10	0.60 ± 0.08	1.09 ± 0.27	1.06 ± 0.17	< 0.001	NS.	NS.

^a^Each value represents Mean ± S.D., n = 10-12. NS., no significant difference.

^b^The significance of differences among four groups were analyzed by two-way ANOVA. Values not sharing a same superscript letters in the horizontal row are significantly different from one another by Duncan’s Multiple Range Test (*P* < 0.05).

^c^Feed efficiency (%) = 〔body weight gain (g)/food intake (g)〕 × 100.

^d^Relative lung weight (%) = (lung weight/body weight) × 100.

### Serum TAS, GSH, and antioxidative enzyme activity

No significant differences in serum TAS, lung GSH, and superoxide dismutase (SOD) activity were observed among all groups (Table 4). The effects of lung catalase, GR, GST, and selenium-dependent glutathione peroxidase (Se-GPx) were significantly reduced in the ALI groups than in the unchallenged groups (*p* < 0.05). The GLN diet had a slight effect on ALI-challenged mice, but this did not reach statistical difference. These results revealed that GLN had no significant benefit on enhancement of antioxidative enzymes.

**Table 4:** **Serum TAS, lung GSH content, and anitixoidative enzyme activity**^**a**^

	**Unchallenged**		**ALI**		***p *** **values from Two-way ANOVA**^**b**^		
	**Control**	**GLN**	**Control**	**GLN**	**ALI**	**GLN**	**ALI × GLN**
Serum TAS (mM)	1.13 ± 0.11	1.11 ± 0.17	1.04 ± 0.07	1.07 ± 0.11	NS.	NS.	NS.
GSH (μmol/g Lung)	4.57 ± 2.44	4.62 ± 2.72	3.89 ± 2.99	6.42 ± 2.52	NS.	NS.	NS.
Catalase (unit/mg protein)	29.1 ± 2.4	29.1 ± 2.5	21.3 ± 2.1	22.3 ± 2.4	< 0.001	NS.	NS.
GR (unit/g protein)	30.6 ± 3.6	31.5 ± 2.6	27.3 ± 3.3	28.5 ± 2.6	< 0.05	NS.	NS.
GST (unit/mg protein)	0.39 ± 0.08	0.41 ± 0.08	0.28 ± 0.04	0.30 ± 0.04	< 0.001	NS.	NS.
SOD (unit/mg protein)	3.07 ± 0.26	3.32 ± 0.24	3.23 ± 0.36	3.28 ± 0.47	NS.	NS.	NS.
Se-GPx (unit/g protein)	44.8 ± 6.4	45.4 ± 9.1	37.4 ± 5.3	43.6 ± 5.4	0.05	NS.	NS.

^a^Each value represents Mean ± S.D., n = 10-12. NS., no significant difference; GSH, glutathione; GR, glutathione reductase; GST, glutathione S-transferase; Se-GPx, se-dependent glutathione peroxidase; SOD, superoxide dismutase; TAS, total antioxidant system.

^b^The significance of differences among four groups were analyzed by two-way ANOVA . Values not sharing a same superscript letters in the horizontal row are significantly different from one another by Duncan’s Multiple Range Test (*P* < 0.05).

### Lung MPO acitivity and IL-1β, IL-6, and TNF-α concentrations

ALI-challenged mice had a significant increase in MPO activity and levels of IL-1β, IL-6, and TNF-α of the lung compared with unchallenged mice (Table 5). The ameliorative effect of the GLN diet was noted for IL-1β and IL-6, and this phenomenon was dependent on ALI-challenge. GLN treatment reduced IL-1β and IL-6 expression by 39% and 69%, respectively, compared with the control group in ALI-challenged mice.

**Table 5:** **Lung MPO acitivity and IL-1β, IL-6, and TNF-α concentrations of mice**^**a**^

	**Unchallenged**		**ALI**		***p *** **values from Two-way ANOVA**^**b**^		
	**Control**	**GLN**	**Control**	**GLN**	**ALI**	**GLN**	**ALI × GLN**
MPO acitivity (unit/g lung)	7.97 ± 13.5	8.27 ± 14.9	207 ± 92	195 ± 92	< 0.001	NS.	NS.
IL-1β (ng/mg protein)	0.02 ± 0.01^c^	0.03 ± 0.02^c^	0.18 ± 0.1^a^	0.11 ± 0.05^b^	< 0.001	NS.	< 0.05
IL-6 (ng/mg protein)	0.11 ± 0.04^c^	0.12 ± 0.05^c^	1.69 ± 1.26^a^	0.56 ± 0.34^b^	< 0.001	< 0.05	< 0.05
TNF-α (ng/mg protein)	0.05 ± 0.02	0.06 ± 0.03	0.26 ± 0.16	0.15 ± 0.17	< 0.001	NS.	NS.

^a^Each value represents Mean ± S.D., n = 10-12. NS., no significant difference.

^b^The significance of differences among four groups were analyzed by two-way ANOVA . Values not sharing a same superscript letters in the horizontal row are significantly different from one another by Duncan’s Multiple Range Test (*p* < 0.05).

### Serum IL-6 and TNF-α concentrations and BALF RAGE, IL-1β, IL-6, and TNF-α levels in ALI-challenged mice

**Figure 1:**
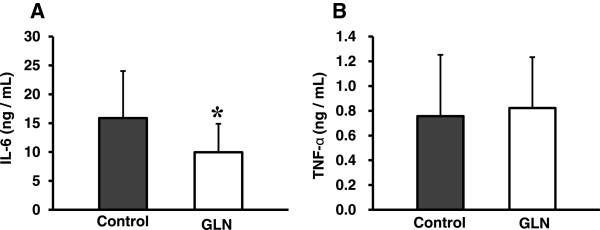
**Serum IL-6 and TNF-α concentrations of ALI-challenged mice. (A)** IL-6, **(B)** TNF-α. Data are presented as mean ± standard deviation; n = 10–12. The effect of GLN was evaluated by Student’s t-test in two ALI-induced groups; (*p < 0.05 vs. the control group).

**Figure 2:**
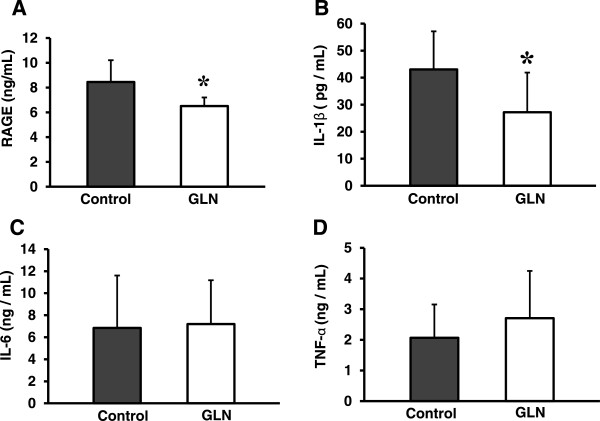
**RAGE concentrations in the BALF of ALI-challenged mice. (A)** RAGE; **(B)** IL-1β; **(C)** IL-6; **(D)** TNF-α. Data are presented as mean ± standard deviation; n = 10–12. The effect of GLN was evaluated by Student’s t-test in two ALI-induced groups (*p < 0.05 vs. the control group).

To examine the anti-inflammatory effect of GLN in ALI-challenged mice, we measured serum cytokine concentrations and used BALF for further analysis. Figure 1 shows that serum TNF-α concentrations did not differ between the control and GLN groups in ALI-challenged mice. In the GLN group, a marked reduction in IL-6 (37%) was observed. As shown in Figure 2, BALF RAGE and IL-1β levels were significantly lower in the GLN group than in the control group of ALI-challenged mice. There were no significant differences in IL-6 and TNF-α levels between the control and GLN groups.

### Lung mRNA expression in ALI-challenged mice

**Figure 3:**
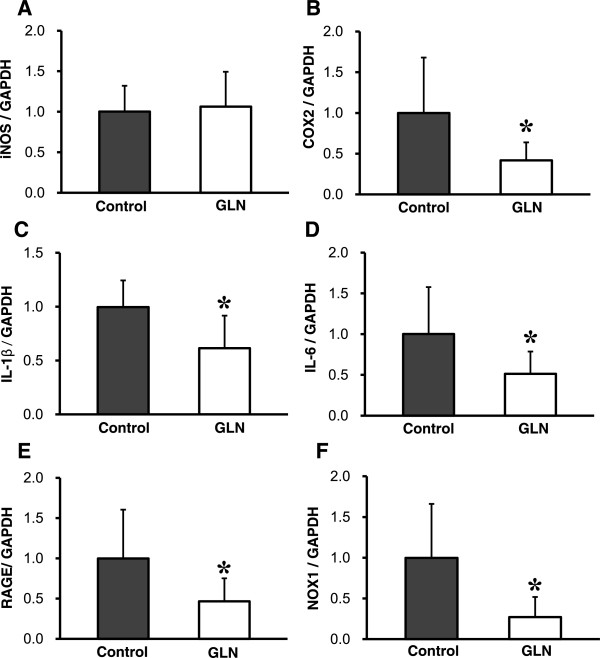
**Relative mRNA levels of inflammatory genes in the lungs of ALI-challenged mice. (A)** iNOS; **(B)** COX-2; **(C)** IL-1β; **(D)** IL-6; **(E)** RAGE; **(F)** NOX-1. Each value was normalized to that of GAPDH, and the relative mRNA abundance was expressed as a fraction of that in the control group and assigned a value of 1. Data are presented as mean ± standard deviation; n = 10–12. The effect of GLN was evaluated by Student’s t-test in two ALI-induced groups (*p < 0.05 vs. the control group).

To further examine the effect of dietary GLN on gene expression, we measured the mRNA levels of inflammatory cytokines, iNOS and COX-2, and the oxidant-generating enzyme, NOX-1. As shown in Figure 3, no statistical difference in iNOS expression was seen between the two groups. GLN treatment reduced COX-2 mRNA expression by 58% compared with the control group. The GLN group had significantly lower mRNA levels of IL-1β and IL-6 compared with the control group (39% and 49%, respectively). RAGE mRNA was significantly decreased in the GLN group. NOX-1mRNA was markedly lower in the GLN group than in the control group.

## Discussion

In our previous studies, we used an animal model of severe ALI by both direct acid and LPS challenge for imitation of clinical cases of direct gastric fluid reflux and bacterial pneumonia. Our previous results indicated that RAGE, TNF-α, and IL-6 expression was significantly higher than that of the unchallenged mice at the early stage (3 and 6 h of injury), but all mice died 48 h after ALI challenge (data not shown). Using this severe ALI model, we speculated that GLN could reduce the expression of these cytokines at an early stage, consequently improving the outcome of ALI. Therefore, the aim of the present study was to determine whether short-term GLN supplementation had a protective effect in the early stages of injury. The dosage of GLN was approximately 0.8 g/kg BW from the feed conversion, a dosage similar to that previously described [25]. As shown in Table 3, enteral GLN administration was safe and did not cause any adverse effects on growth. The survival rate 24 h after ALI challenge was 33% in the GLN group and 11% in the control group (data not shown). The ameliorative effect of GLN supplementation on the survival rate was not complete, which may be a result of more severe injury in this model of ALI induced by two powerful chemicals. A study conducted by Zhou *et al*. [26] demonstrated that IP LPS injection plus HCl aspiration produced significant lung injury, compared with either LPS injection or HCl instillation alone. Therefore, dietary GLN supplementation had a beneficial effect only within 24 hours after this severe ALI challenge, but in the end could not rescue survival. We will try to modify this severe ALI model by reducing the dose of LPS/acid in next study to further explore the effect of dietary GLN on survival.

To elucidate the anti-inflammatory effect of GLN in ALI-challenged mice, we did not analyze BALF cytokines and lung mRNA expression in both unchallenged groups because we found that the levels of these cytokines of unchallenged controls were very low or undetectable in our previous study (data not shown). The other reason was to reduce this experimental cost and assay load, which caused this experimental design flaw in our studies. Therefore, the possible effects of GLN discussed below focus on the ALI-challenged condition in mice.

There are a number of possible explanations for the protective effect of GLN against ALI during sepsis, including enhanced expression of heat shock protein (HSP), activation of peroxisome proliferator-activated receptor (PPAR)-γ, buffering of oxidative stress, inhibition of HMGB-1 expression, and anti-inflammatory responses [13,15]. This model of acid and LPS introduced into the trachea led to a direct, chemically induced, tissue-level injury of the lung in such a short time that there was no immediate mounting of an immune response, as is observed in the normal model of septic system injury. Given our results, we speculated that the key function of GLN was to reduce the inflammatory response by inhibiting RAGE expression and pro-inflammatory cytokine production.

First, as shown in Figures 2A and 3E, this study demonstrated that dietary GLN could downregulate RAGE expression in the lung. RAGE is a marker of type I cell injury in ALI [8]. A previous report indicated that HMGB-1 was a late mediator of endotoxin-induced ALI and an early trigger of inflammation in animal models via RAGE activation [27]. Administration of recombinant RAGE could attenuate injury in LPS-induced ALI in mice [7]. Recently, BALF RAGE was considered a sensitive indicator for direct lung injury in acid or LPS-induced ALI in mice, particularly in acid-challenged mice [7]. Yamakawa and colleagues [28] indicated that LPS stimulation resulted in release of RAGE into the media in a dose-dependent manner from cultured rat alveolar epithelial cells, and verified the that proteolysis was primarily caused by matrix metalloproteinase (MMP)-3 and -13. Therefore, we also measured MMP-3 mRNA in the lung. The GLN diet decreased relative MMP-3 mRNA expression by 30%, but this did not reach statistical difference (data not shown). Confirmation of whether the mechanism by which GLN treatment causes a decrease in RAGE expression is mediated by proteolysis is beyond the scope of this study. According to previous reports, attenuation of RAGE activation led to a decrease in ALI severity. Thus, we suggest that dietary GLN exerted its protective role by decreasing RAGE expression in ALI-challenged mice.

Second, we found that lung IL-1β and IL-6 levels were significantly lower in the GLN group with corresponding decreases in their mRNA expression than was observed in the control group. We speculate that proinflammatory cytokines such as TNF-α, IL-1β, and IL-6 were released in the early stage of ALI challenge; in other studies, IL-6 was considered a particularly useful marker for prediction of the severity of sepsis. Previous reports revealed that enteral GLN supplementation for 3 weeks decreased lung IL-6 levels and regulated the immune response during CLP-induced sepsis [29,30]. Thus, we propose that GLN supplementation had an anti-inflammatory effect against ALI challenge at an early stage.

Third, worsening oxidative stress was a possible cause of ALI damage. To determine whether the antioxidant properties of GLN are associated with ALI-induced oxidative stress, we measured GSH and antioxidative enzymes. The lung GSH concentration and mRNA level of γ-glutamyl-cysteine-synthetase (data not shown) were unchanged in the GLN group. Recent experimental data have demonstrated that HSP70 enhancement is the main factor responsible for the beneficial effects of GLN on ALI during sepsis, because GLN is required for HSP generation [15,25,31]. It is unclear whether short-term dietary GLN supplementation is sufficient to generate the level of HSP required for buffering the acid and LPS-induced damage. Therefore, Hsp70 expression warrants further investigation. Interestingly, we found that mice that received GLN supplementation showed a marked reduction in lung COX-2 and NOX-1 mRNA. A previous study showed that lung COX-2 expression and activity were increased in acid-induced injury, and the actual role of COX-2 varied at different stages of injury [32]. NOX-1 has been identified as the major oxidant-generating enzyme in neutrophils and macrophages [33]. A previous report demonstrated that NOX-1 was responsible for more than 90% of the lipid-derived free radicals and was implicated in lung damage in an IT LPS-induced lung injury model in rats [34]. Lung injury and neutrophil infiltration were attenuated in NOX-1 null mice (deficient in the gp91^phox^ and p47^phox^ subunits of Nox) [35,36]. NOX-1 inhibitors have been considered a potential therapy for ALI [34]. Therefore, NOX-1 inhibition might have an additional benefit in GLN-treated mice after ALI challenge.

MPO is a useful marker of neutrophil infiltration in different ALI models [37,38]. Our data showed that 3 h of ALI challenge resulted in significantly higher MPO activity than in unchallenged mice, but no abrogation effect was caused by GLN treatment. It is possible that the selected time point was not appropriate for GLN function, thus the decreased effect on MPO activity was not observed in our study, unlike previous reports [39,40]. On the other hand, toll-like receptor 4 (TLR4) signaling was believed to be another pathway of acid and LPS-induced ALI other than RAGE expression. In mice treated with adenovirus-mediated siRNA targeting the TLR4 gene, a protective effect was shown against LPS challenge [41]. Thus, whether TLR4 signaling was involved in the beneficial effects of GLN remains to be explored in future studies.

## Conclusions

Our results suggest that the beneficial effects of dietary GLN supplementation might be partially attributed to an inhibitory effect on RAGE expression and pro-inflammatory cytokine production at an early stage in acid- and LPS-induced ALI.

## Abbreviations

ALI: Acute lung injury; ARDS: Acute respiratory distress syndrome; BALF: Bronchoalveolar lavage fluid; CLP: Cecal ligation and puncture; COX-2: Cyclooxygenase-2; GLN: Glutamine; GAPDH: Glyceraldehyde-3-phosphate dehydrogenase; GSH: Glutathione; GPx: Glutathione peroxidase; GR: Glutathione reductase; GST: Glutathione S-transferase; HSP: Heat shock protein; HMGB-1: High-mobility group box protein-1; iNOS: Inducible nitric oxide synthase; IT: Intratracheally; LPS: Lipopolysaccharide; MMP: Metalloproteinase; MPO: Myeloperoxidase; NOX-1: NADPH oxidase-1; PMS: Post-mitochondrial fraction; PPAR: Peroxisome proliferator-activated receptor; RAGE: Receptor for advanced glycation end-products; SOD: Superoxide dismutase; TAS: Total antioxidant system; TLR-4: Toll-like receptor 4; TNF-α: Tumor necrosis factor-α.

## Competing interest

The authors declare that they have no conflict of interest.

## Authors’ contributions

Y-CC and C-CC provided the ALI model and technical guidance. H-MS conducted the analysis of antioxidative enzymes. H-JP and W-CL performed the experiments. H-LH designed and integrated this study, and drafted and revised the manuscript. All authors read and approved the final manuscript.

## Pre-publication history

The pre-publication history for this paper can be accessed here:

http://www.biomedcentral.com/1471-2466/14/115/prepub
